# Folic Acid Improves the Inflammatory Response in LPS-Activated THP-1 Macrophages

**DOI:** 10.1155/2018/1312626

**Published:** 2018-07-04

**Authors:** Mirian Samblas, J. Alfredo Martínez, Fermín Milagro

**Affiliations:** ^1^Department of Nutrition, Food Science, and Physiology, Centre for Nutrition Research, University of Navarra, Pamplona, Spain; ^2^Centro de Investigación Biomédica en Red de la Fisiopatología de la Obesidad y Nutrición (CIBERobn), Instituto de Salud Carlos III, Madrid, Spain; ^3^Instituto de Investigación Sanitaria de Navarra (IdiSNA), Pamplona, Spain; ^4^IMDEA Food, Madrid, Spain

## Abstract

DNA methylation has been suggested as a regulatory mechanism behind some inflammatory processes. The physiological actions of methyl donors, such as folic acid, choline, and vitamin B_12_ on inflammation-related disease have been associated with the synthesis of the universal methyl donor S-adenosyl methionine (SAM). The aim of this study was to evaluate the effects of folic acid, choline, vitamin B_12_, and a combination of all on preventing the lipopolysaccharide- (LPS-) induced inflammatory response in human THP-1 monocyte/macrophage cells. Folic acid and the mixture of methyl donors reduced interleukin 1 beta *(IL1B)* and tumour necrosis factor *(TNF)* expression as well as protein secretion by these cells. Folic acid and choline decreased C-C motif chemokine ligand 2 (*CCL2*) mRNA levels. In addition to this, the methyl donor mixture reduced Cluster of differentiation 40 *(CD40)* expression, but increased serpin family E member 1 *(SERPINE1)* expression. All methyl donors increased methylation levels in CpGs located in *IL1B*, *SERPINE1*, and interleukin 18 *(IL18)* genes. However, *TNF* methylation was not modified. After treatment with folic acid and the methyl donor mixture, ChIP analysis showed no change in the binding affinity of nuclear factor-*κ*B (NF-*κ*B) to *IL1B* and *TNF* promoter regions after the treatment with folic acid and the methyl donor mixture. The findings of this study suggest that folic acid might contribute to the control of chronic inflammation in inflammatory-related disease.

## 1. Introduction

Inflammation is traditionally defined as the short-term adaptive response to fight against injury, caused by pathogens or biological and chemical stimuli [1]. Although acute inflammation is a crucial component for maintaining homeostasis in the body, persistent and chronic inflammation is involved in the development of several clinical manifestations and diseases. During inflammatory disease, monocytes and macrophages produce cytokines in response to different stimuli, such as lipopolysaccharide (LPS) [2]. The proinflammatory molecules released by macrophages in the inflamed regions orchestrate the enhancement of monocyte recruitment from blood to tissue. Recruited monocytes differentiate into macrophages to continue the inflammatory response [3]. Alongside this, studies have described that epigenetic mechanisms contribute to the pathogenesis of several chronic inflammatory-related diseases by regulating important steps such as macrophage infiltration or cytokine secretion [4, 5]. For instance, inflammatory genes like interleukins (IL) *IL6*, *IL4*, *IL8*, *IL1B*, or interferon *INF*-*γ* have been described to be methylated differently in several chronic inflammatory diseases [4, 6].

Methionine, folate, betaine, choline, and vitamins B_2_, B_6_, and B_12_ are considered methyl donor precursors naturally occurring in the diet [7]. These substances participate in the methionine pathway for the synthesis of S-adenosyl methionine (SAM), which is the universal methyl donor for DNA methylation reactions [8]. The anti-inflammatory effects exerted by some of these compounds in a variety of inflammatory diseases have been described in previous research. For example, folic acid supplementation improved disease outcomes in subjects with hypertension, diabetes, and stroke by reducing levels of inflammatory markers (CRP, VCAM-1, IL-1*β*, and TNF-*α*) [9]. In addition to this, the combined supplementation of folate and vitamin B_12_ ameliorated inflammation during pregnancy by modifying the concentration of inflammatory cytokines [10]. Lastly, vitamin B_12_ has been negatively associated with proinflammatory cytokines and low-grade systemic inflammation [11]. On the other hand, deficiencies in the abovementioned methyl donors have also been shown to lead to adverse effects [10, 12]. For example, vitamin B_12_ deficiency has been related to metabolic disturbances such as hyperhomocysteinemia, obesity, hypertension, and insulin resistance [10]. Choline deficiency has been associated with the development of fatty liver disease and demonstrated to worsen the outcome of liver fibrosis in patients with nonalcoholic steatohepatitis (NASH) [12]. Interestingly, NASH patients that were choline-deficient exhibited amelioration of steatohepatitis after choline supplementation [13]. Similarly, methyl donor supplementation prevented HFS diet-induced liver fat accumulation in rats fed an obesogenic diet [14].

The anti-inflammatory effect of methyl donors in monocytes before differentiation and LPS-induced inflammatory response in macrophages, along with the role of DNA methylation in this process, have been scarcely studied. For this reason, the aim of this study was to investigate the effects of methyl donors, both individually and together, on the attenuation of LPS-induced inflammatory response and the possible underlying epigenetic mechanisms in human THP-1 monocyte/macrophage cells. Monocytes were incubated with folic acid, choline, and vitamin B_12_ or a methyl donor mixture of folic acid, choline, and vitamin B_12_. Monocytes were then differentiated into macrophages and an inflammatory response was induced with LPS.

## 2. Material and Methods

### 2.1. Reagents

Folic acid, vitamin B_12_, and choline chloride were supplied from Sigma-Aldrich (MO, USA). Phorbol 12-myristate 13-acetate (TPA) (Sigma-Aldrich) was used for differentiating THP-1 monocytes into macrophage-like cells. LPS from *E. coli* K12 strain (Invitrogen, CA, USA) was applied to activate macrophages. Thiazolyl Blue Tetrazolium Bromide (MTT) (Sigma-Aldrich) was used to investigate the toxic effects of methyl donors on THP-1 cells.

### 2.2. Cell Culture and Treatments

Human monocyte THP-1 cells were purchased from American Type Cell Culture (ATCC® TIB-202™, VA, USA). Cells were maintained at 37°C and 5% CO_2_ in RPMI-1640 medium (Gibco) modified to contain 2 mM L-glutamine, 1 mM sodium pyruvate, 4.5 g/l glucose, and 1.5 g/l sodium bicarbonate and supplemented with 10% fetal bovine serum (GIBCO), 100 U/ml penicillin, and 100 *μ*g/ml streptomycin.

THP-1 cells were treated with 11.3 *μ*M folic acid, dissolved in 1 M NaOH, 105 *μ*M choline chloride, 18.5 nM vitamin B_12_, and a mixture of methyl donors consisting of folic acid, choline chloride, and vitamin B_12_ at the concentrations previously indicated. Concentrations were determined by multiplying ten times the basal concentration present in the RPMI-1640 medium for each compound. After 24 h, cells were differentiated into macrophages by incubation with 25 ng/ml TPA for 48 h and then were activated with 100 ng/ml LPS for 24 hours. Finally, RNA and DNA were extracted and supernatants were collected for ELISA analysis.

### 2.3. Cell Viability Analysis

For the viability assay, THP-1 cells were pretreated with methyl donors at the selected concentrations during 24 h, as described above, in a 96-well plate. After the treatments, 20 *μ*l MTT (5 mg/ml) was added to each well and plates were incubated for 2 h at 37°C. Formazan crystal formation was solubilised in 100 *μ*l/well DMF-glacial acetic acid-SDS solution consisting of 40% DMF, 2% glacial acetic acid, and 16% *w*/*v* sodium SDS. Formazan production was quantified by absorbance at 570 nm using a microplate reader (Multiskan Spectrum, Thermo Electron Corporation, Finland). The results were expressed as relative cell viability (%).

### 2.4. Analysis of mRNA Expression by Quantitative Real-Time PCR

Total RNA was extracted from cells with TRizol® Reagent (Invitrogen). RNA quality and concentration were measured using the Nanodrop Spectrophotometer ND1000 (Thermo Fisher Scientific, MA, USA). Approximately 1 *μ*g of total RNA was reverse-transcribed into cDNA by the MultiScribe™ Reverse Transcriptase Kit, following the manufacturer's instructions (Thermo Fisher Scientific, MA, USA). Real-time PCR was performed using ABI Prism 7900HT Sequence Detection System and Taqman Universal Master Mix (Applied Biosystems, CA, USA). Predesigned TaqMan primers and probes for *IL1B* (Hs01555410_m1), *TNF* (Hs00174128_m1), *IL18* (Hs01038788_m1), *SERPINE1* (Hs01126606_m1), *CD40* (Hs01002913_g1), *CCL2* (Hs00234140_m1), and *TLR4* (Hs00152939_m1) genes were used (Applied Biosystems). The levels of these mRNAs were normalized to the level of *GAPDH* (Hs02758991_g1) mRNA expression. Relative expression was determined by using the comparative 2^−ΔΔCt^ method.

### 2.5. Cytokine Secretion Analysis by Enzyme-Linked Immunosorbent Assay (ELISA)

Culture supernatants were collected after the treatments and stored at −80°C for further cytokine analysis. Protein concentrations of IL-1*β*, TNF-*α*, PAI1, and CD40 were measured with standard ELISA kits (R&D Systems Europe, UK), according to the manufacturer's protocols. Absorbance was measured at 450 nm using a microplate reader (Multiskan Spectrum, Thermo Electron Corporation, Finland).

### 2.6. DNA Methylation Analysis by MALDI-TOF Mass Spectrometry

DNA was isolated using MasterPure™ DNA Purification Kit (Illumina, WI, USA), according to the manufacturer's guidelines. Genomic DNA was sodium bisulfite-converted using the EpiTect Bisulfite Kit (Qiagen, CA, USA). DNA methylation quantification was performed by MassARRAY EpiTYPER technology (Sequenom Inc., CA, USA). This method uses matrix-assisted laser desorption ionization time-of-flight (MALDI-TOF) mass spectrometry in combination with RNA base-specific cleavage (MassCLEAVE). Four amplicons covering 32 CpG sites were selected. EpiDesigner software (Sequenom Inc.; http://www.epidesigner.com/start3.html) was used to design PCR primers for the amplicons of interest: *IL1B* (chr2: 112,837,566–112,837,895), *TNF* (chr6: 31,575,209–31,575,481), *SERPINE1* (chr7: 101,127,068–101,127,411), and *IL18* (chr11: 112,163,853–112,164,105). The designed primers are shown in Supplementary Table 1 and the complete amplicon sequences are reported in Supplementary Figure 1. The complete methodology was previously explained [15].

### 2.7. Chromatin Immunoprecipitation (ChIP) Assay

ChiP assay was performed with the ChIP-IT™ Express Enzymatic Kit (Active Motif, CA, USA), following the manufacturer's guidelines. THP-1 cells were cultured for 24 hours with methyl donors and were then differentiated with TPA (25 ng/*μ*l) for 48 hours and activated with LPS (100 ng/*μ*l) over 24 hours. The cell medium was discarded and 36.5% formaldehyde was added directly to the cell surface for 10 min. Cross-linking between proteins and DNA was stopped by the addition of glycine for 5 min at room temperature and cells were collected by scraping. Then, cells were incubated with lysis buffer for 30 min at 4°C and DNA was fragmented via enzyme-based digestion for 10 min at 37°C. Chromatin was immunoprecipitated using rabbit polyclonal antibody to nuclear factor-*κ*B (NF-*κ*B; ab7970, Abcam, MA, USA). After immunoprecipitation, cross-linking of protein-DNA complexes was reversed. Real-time quantitative PCR was performed using primers for *IL1B*: sense 5′-agcaacaaagctgccactta-3′ and antisense 5′-tgacgtgctgtgtgaatttg-3′, and *TNF*: sense 5′-ggagaatgtccagggctatg-3′ and antisense 5′-tcctggaggctctttcactc-3′.

### 2.8. Transcription Factor-Binding Site Analysis

In order to identify the putative transcription factor binding site in the CpG sites of the *IL1B* gene, a bioinformatic analysis was performed through LASAGNA-Search 2.0 using TRANSFAC matrices and aligned models, as described elsewhere [16].

### 2.9. Statistical Analysis

Normality was assessed by Kolmogorov-Smirnov and Shapiro-Wilk tests. For the statistical analysis of the results, a one-way ANOVA followed by Dunnett's test for multiple comparisons between groups and an unpaired Student *t*-test, for the direct comparisons between two groups, were used. Differences were considered significant at *P* value < 0.05. Statistics were performed using Prism 5.0 (GraphPad Software, CA, USA).

## 3. Results

### 3.1. Methyl Donors Did Not Affect Cell Viability

Cell viability was measured by MTT assay after incubation with folic acid at 11.3 *μ*M, choline at 105 *μ*M, vitamin B_12_ at 18.5 nM, and the methyl donor mixture. The selected concentrations were within the range proposed by previous studies [9, 17, 18]. Cell viability was not significantly affected by methyl donors at these concentrations (Supplementary Figure 2).

### 3.2. Effects of Methyl Donors on the Expression of Genes Associated with the Inflammatory Response in THP-1 Macrophages Activated with LPS

**Figure 1:**
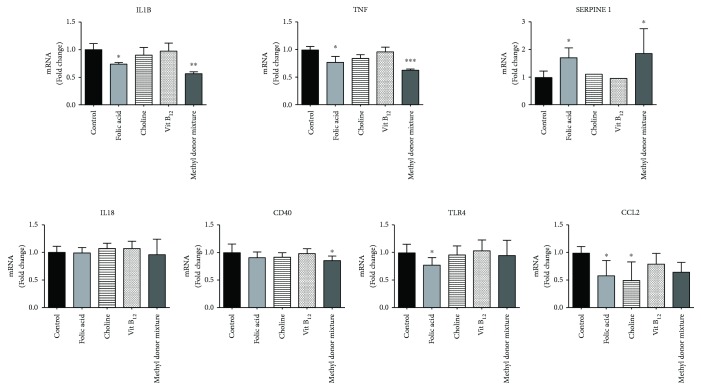
Effects of folic acid, choline, vitamin B_12_, and the methyl donor mixture on the expression of inflammatory genes in THP-1 cells treated with TPA and LPS. Results are expressed as means ± SD (*n* = 8). Differences between groups were analyzed by one-way ANOVA followed by Dunnett's test. ^∗^ *P* value < 0.05, ^∗∗^ *P* value < 0.01, and ^∗∗∗^ *P* value < 0.001 versus control.

Treatment of THP-1 cells with the different compounds before the differentiation with TPA and activation with LPS altered the expression of most of the inflammation-related genes compared to the control treatment (Figure 1). Folic acid and the methyl donor mixture reduced *IL1B* (*P* < 0.05 for folic acid; *P* < 0.01 for the methyl donor mixture) and *TNF* (*P* < 0.05 for folic acid; *P* < 0.001 for methyl donor mixture) mRNA expression. Folic acid also reduced *TLR4* (*P* < 0.05) and *CCL2* (*P* < 0.05), but increased *SERPINE1* (*P* < 0.05) gene expression. Moreover, methyl donor mixture incubation reduced the levels of *CD40* (*P* < 0.05) but increased *SERPINE1* (*P* < 0.05). Choline decreased the expression of *CCL2* (*P* < 0.05). However, no statistically significant changes were observed after vitamin B_12_ incubation.

### 3.3. The Pretreatment with Folic Acid and Methyl Donor Mixture Reduced IL-1*β* and TNF-*α* Secretion of LPS-Activated Macrophages

**Figure 2:**
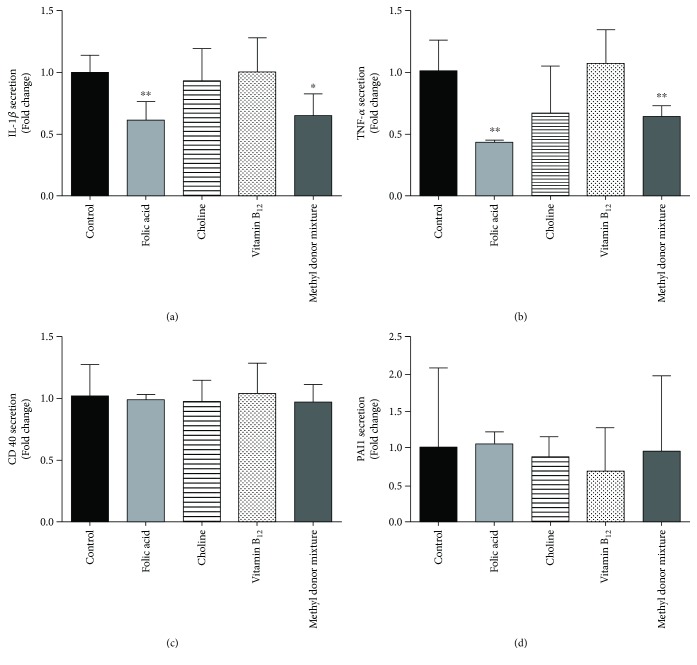
Effects of folic acid, choline, vitamin B_12_, and the methyl donor mixture on the secretion of inflammatory cytokines in THP-1 cells treated with TPA and LPS. (a) IL-1*β*, (b) TNF-*α*, (c) CD40, and (d) PAI-1 secretion. Results are expressed as means ± SD (*n* = 7–8). Differences between groups were analyzed by one-way ANOVA followed by Dunnett's test. ^∗^ *P* value < 0.05 and ^∗∗^ *P* value < 0.01.

Concerning cytokine secretion, the incubation with folic acid and the methyl donor mixture reduced the levels of IL-1*β* (*P* < 0.01) and TNF-*α* (*P* < 0.01 and *P* < 0.05, resp.), but not CD40 and PAI-1. However, no changes were observed with the other methyl donors (Figure 2).

### 3.4. Incubation with Methyl Donors Increased DNA Methylation in the Inflammatory Genes

The regions studied in the genes *IL1B*, *SERPINE1*, and *IL18* displayed an overall gain of methylation when LPS-activated macrophages were treated with the different methyl donors. This hypermethylation was especially significant after the incubation with folic acid. As shown in Table 1, folic acid significantly increased (*P* < 0.05) the methylation levels of CpG_1 (190%), CpG_5 (680%), and CpG_6 (200%) of *IL1B*, CpG_1 (750%), CpG_2 (88%), CpG_3.4 (136%), CpG_7 (1003%), and CpG_9 (88%) of *SERPINE1*, and CpG_4 (53%) and CpG_5 (27%) of *IL18* when compared with the methylation percentage of the nontreated LPS-activated macrophages. No changes in methylation were noted in the analyzed region of *TNF* after the treatment. Regarding choline chloride, vitamin B_12_, and the methyl donor mixture, incubation with these compounds also significantly increased (*P* < 0.05) the methylation levels of some CpG sites concerning the studied genes (Table 1).

### 3.5. NF-*κ*B Binding to IL-1*β* and TNF-*α* Was Not Affected after the Incubation with Folic Acid and the Methyl Donor Mixture

**Figure 3:**
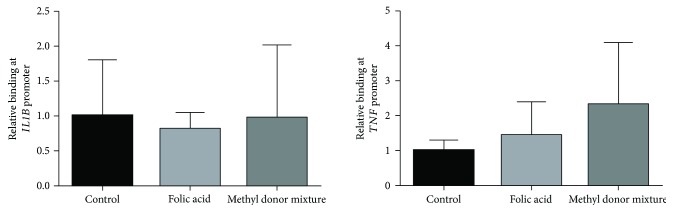
Relative binding of NF-*κ*B to *IL1B* and *TNF* promoters in THP-1 cells differentiated with TPA and activated with LPS (control group) and the same cells treated with folic acid and the methyl donor mixture before differentiation. Results are expressed as means ± SD (*n* = 8). Differences between groups were tested by one-way ANOVA.

*IL1B* and *TNF* gene expression and secretion decreased after the incubation with folic acid and the methyl donor mixture (Figures 1 and 2). *IL1B* DNA methylation levels increased; *TNF* methylation levels did not. To determine the effect of DNA methylation in the sequence of proinflammatory genes on NF-*κ*B binding to *IL1B* and *TNF* promoters, a ChIP assay was performed. The analysis showed no significant changes in NF-*κ*B binding to *IL1B* and *TNF* promoter regions in THP-1 cells treated with folic acid and the methyl donor mixture (Figure 3).

## 4. Discussion

Previous studies in humans have analyzed the association between folic acid and inflammation. For example, a case-control study showed a reduction of cytokine levels after a 12-week treatment with folic acid [19]. In addition, folic acid supplementation in patients with a high risk of coronary artery disease was associated with a reduction in proinflammatory cytokines (e.g., monocyte chemoattractant protein 1 or MCP-1) in human monocytes [20]. In the present study, we demonstrated that folic acid and a mixture of methyl donors reduced the expression of proinflammatory genes (e.g., *TNF*, *IL1B*, *CD40*, *CCL2*, and *TLR4*) in THP-1 monocytes, when the monocytes were differentiated into macrophages and activated with LPS. In agreement with our results, the incubation of murine monocyte RAW 264.7 cells with folic acid reduced the expression of proinflammatory genes during LPS activation [17] . In contrast, folic acid deficiency in the same cell line enhanced the expression of proinflammatory genes [21].

Current data revealed that folic acid and the methyl donor mixture not only reduce proinflammatory gene expression in THP-1 monocytes, but also decrease the secretion of TNF-*α* and IL-1*β* cytokines when cells were differentiated to macrophages and activated by LPS.

During the inflammatory response, the proinflammatory mediators, specially MCP-1 (encoded by the *CCL2* gene), contribute to the migration of circulatory monocytes into the surrounding tissue [22], by specifically attracting monocytes towards the inflamed area, promoting tissue damage and disease. In this context, our results suggest that folic acid and a mixture of methyl donors could reduce the inflammatory response of the monocytes and the macrophages derived from these monocytes would decrease cytokine and chemokine secretion.

The specific mechanisms for the beneficial effects of folic acid or methyl donors on inflammation have not been clearly elucidated. One of the possible explanations is epigenetics, via DNA and histone methylation [23]. Folate, choline, and vitamin B_12_ directly participate in the formation of S-adenosyl methionine (SAM), which is the major donor of methyl groups for DNA methylation [24]. In the current trial, folic acid and the methyl donor mixture increased methylation levels of *IL1B*, *SERPINE1*, and *IL18.* However, only *IL1B* presented lower gene expression and protein secretion, which was associated with hypermethylation after folic acid supplementation. This result suggests that *IL1B* gene expression may be modulated by changes in DNA methylation induced by folic acid. However, the methylation changes of *SERPINE1* and *IL18* did not correlate with changes in gene expression. Incubation with methyl donors was for 24 hours and then the monocytes were differentiated for 48 hours and activated by LPS for a further 24 hours. A recent study of *IL18* expression in LPS-stimulated murine macrophages showed that the maximum level of expression of this interleukin was 3–6 hours after induction. No changes were found after 24 hours suggesting an earlier enzymatic activation of poly(ADP-ribose) polymerase 1 (PARP-1) that induces *IL18* expression [24]. In this context, the time or the concentration of supplements might have been insufficient to evidence subtle changes in *IL18* and *SERPINE1* expression.

Unexpectedly, no changes in DNA methylation levels in the *TNF* gene were found after the treatment with methyl donors. In line with these results, Kolb and Petrie [21] found that although folate deficiency in murine macrophages reduced DNA methyltransferase expression, *TNF* DNA methylation did not change. In addition, in murine macrophages incubation with exogenous SAM attenuated the LPS-stimulated expression of TNF [25]. The reduction in *TNF* expression without a change in methylation could be due to folic acid causing upstream methylation. Recent studies have uncovered a new epigenetic mechanism for gene expression regulation: mRNA methylation. *In vitro* data has shown that methyladenine of mRNA influences mRNA transcription, splicing, nuclear export, translation, and mRNA stability [26, 27]. Therefore, RNA methylation may also be a regulatory mechanism altering *TNF* expression after the supplementation with the methyl donors.

Transcriptional activation of *TNF* and *IL1B* by LPS requires the stimulation of a set of pathways and transcription factors, including NF-*κ*B, early growth response protein 1 (EGR-1), and activator protein 1 (AP-1) [28]. Available data in such research reveals that the binding levels of the NF-*κ*B transcription factor to the *IL1B* promoter were similar to the *TNF* promoter and to macrophages without the methyl donors' supplementation. These results suggest that DNA methylation does not directly affect the binding affinity of NF-*κ*B to *IL1B*. Similarly, Feng et al. [17] reported that folic acid decreased TNF-*α* and IL-1*β* production by inhibiting the NF-*κ*B pathway without modifying NF-*κ*B binding affinity. Taking into account these results, other molecular mechanisms could be affected by the tested molecules. For example, the bioinformatic analysis of the selected sequence of *IL1B* identified a putative PU.1 (Spi-1) transcription factor binding site, which could be involved in the regulation of the expression of this gene. PU.1 binds to GC-rich regions of genes to activate transcription, hence DNA methylation might impair the binding of PU.1 to the analyzed sequence and downregulate gene transcription. Interestingly, the PU.1 transcription factor is involved in macrophage differentiation and also in the transcriptional control of genes in mature macrophages [29, 30].

THP-1 is a human monocytic cell line derived from peripheral blood, which has been widely used to investigate the inflammatory response due to its ability to differentiate into macrophage-like cells. A known limitation of the use of cell lines in research is the difference to the natural environment, however previous studies have found that LPS mimics the inflammatory environment when added to THP-1 [31]. Despite this, direct extrapolation to human disease is not possible because more proinflammatory molecules and more than one cell type are involved in the response. Nevertheless, results of this investigation suggest a direct effect of methyl donors in the methylation of proinflammatory genes in LPS-activated THP-1 cells and the reduction of expression and production of proinflammatory cytokines. In addition, although methyl donor supplementation did not modify *TNF* promoter methylation, it reduced LPS-induced TNF-*α* production. However, the binding affinity of NF-*κ*B to proinflammatory genes was unaffected, suggesting a minor role of this protein complex in the transcriptional regulation of these genes in response to folic acid and other methyl donors.

## 5. Conclusion

The findings of this study evidenced that monocyte pretreatment with specific methyl donors, particularly folic acid, reduced the inflammatory response in LPS-activated THP-1 macrophages, which could in part be mediated by increased DNA methylation in some CpG sites of important proinflammatory genes. In addition, folic acid decreased the expression of cytokines and chemokines (i.e., CCL2), suggesting a protective role through the recruitment of monocytes to the inflamed tissue.

## Figures and Tables

**Table 1:** CpG methylation levels (as percentage) in *IL1B*, *TNF*, *IL18*, and *SERPINE1* genes after the incubation of THP-1 monocytes with folic acid, choline, vitamin B_12_, and the methyl donor mixture, measured by MassARRAY. Unpaired Student's *t*-test was used to compare each CpG with the control group. ^∗^ *P* value < 0.05; ^∗∗^ *P* value < 0.01; and ^∗∗∗^ *P* value < 0.001.

	Control	Folic acid	Choline	Vitamin B_12_	Methyl donor mixture
*IL1B*					
CpG_1	6.88 ± 3.97	15.5 ± 6.14^∗^	11.4 ± 1.55^∗^	9.38 ± 0.95	14.0 ± 3.11^∗^
CpG_2	94.9 ± 3.14	96.2 ± 1.66	94.8 ± 2.06	9.76 ± 0.85	96.9 ± 0.85
CpG_3	2.88 ± 2.09	1.75 ± 1.26	5.75 ± 1.77	3.75 ± 3.89	3.12 ± 3.75
CpG_4	2.75 ± 2.06	1.88 ± 1.79	1.25 ± 1.19	1.75 ± 0.96	3.75 ± 1.55
CpG_5	2.12 ± 2.49	6.50 ± 4.06^∗^	1.25 ± 0.64	3.25 ± 1.55	10.0 ± 1.13
CpG_6	0.75 ± 0.50	7.12 ± 6.14^∗^	9.50 ± 0.58^∗∗∗^	9.25 ± 2.33^∗∗∗^	0.88 ± 0.48

*TNF*					
CpG_1	98.2 ± 1.32	96.1 ± 2.78	98.0 ± 1.47	95.2 ± 2.72	95.8 ± 3.07
CpG_2	67.8 ± 3.95	64.8 ± 7.59	65.8 ± 4.48	69.9 ± 1.93	63.4 ± 6.46
CpG_3	48.1 ± 12.6	48.6 ± 8.53	51.2 ± 10.3	47.9 ± 4.71	45.0 ± 4.65
CpG_4.5.6	19.9 ± 6.76	15.2 ± 4.41	17.4 ± 3.09	19.8 ± 0.87	17.8 ± 1.79
CpG_8	33.9 ± 3.49	29.6 ± 4.37	31.8 ± 3.93	34.2 ± 2.53	34.0 ± 3.24

*IL18*					
CpG_1	12.5 ± 17.4	2.0 ± 0.5	2.12 ± 0.85^∗^	1.25 ± 1.32	2.5 ± 2.0
CpG_2	6.88 ± 1.55	6.0 ± 1.22	7.38 ± 2.06^∗^	9.50 ± 1.0	10.1 ± 2.25
CpG_3	0.67 ± 0.29	2.0 ± 0.82	0.62 ± 0.25	1.38 ± 0.75	0.88 ± 0.75
CpG_4	11.0 ± 2.04	8.38 ± 4.09^∗^	7.75 ± 2.22	8.0 ± 1.91	7.25 ± 1.85
CpG_5	19.0 ± 3.19	13.2 ± 2.10^∗^	15.8 ± 2.90	16.6 ± 1.11	15.0 ± 2.16

*SERPINE1*					
CpG_1	2.62 ± 0.63	22.0 ± 13.7^∗∗^	10.8 ± 14.2	17.0 ± 16.1^∗^	56.2 ± 5.14
CpG_2	33.5 ± 3.39	63.0 ± 12.5^∗∗∗^	50.8 ± 7.09^∗∗^	65.0 ± 5.40^∗∗∗^	45.4 ± 8.68^∗^
CpG_3.4	38.5 ± 9.81	91.1 ± 8.23^∗∗^	79.5 ± 7.99^∗∗∗^	85.2 ± 10.9^∗∗∗^	62.6 ± 24.0^∗^
CpG_6	100 ± 0.00	89.4 ± 4.09	90.9 ± 11.3	87.1 ± 10.2	96.6 ± 5.49
CpG_7	2.88 ± 1.60	32.1 ± 15.4^∗∗^	13.9 ± 7.97^∗^	28.2 ± 23.1^∗^	15.8 ± 15.8^∗^
CpG_8	95.1 ± 2.62	91.5 ± 6.77	92.6 ± 6.74	93.1 ± 2.66	94.9 ± 2.06
CpG_9	33.5 ± 3.39	63.0 ± 12.5^∗∗^	50.8 ± 7.09^∗∗^	65.0 ± 5.40^∗∗∗^	45.4 ± 8.68
CpG_10	97.2 ± 2.59	95.0 ± 3.03	96.6 ± 1.60	88.5 ± 19.7	96.9 ± 2.46
CpG_11	97.5 ± 2.91	98.2 ± 2.36	97.1 ± 2.69	98.1 ± 0.75	98.5 ± 2.68
CpG_12	94.0 ± 7.22	95.0 ± 2.42	94.2. ± 5.52	96.0 ± 3.58	92.4 ± 6.26

## Data Availability

Access to data will be considered by the author upon request.

## Supplementary Materials

Supplementary Materials Supplementary Table 1: primer sequences used for MassARRAY EpiTYPER assay. Supplementary Figure 1: genomic localization and nucleotide sequences of CpG sites covered by the MassARRAY EpiTYPER probes for the study of DNA methylation levels of *IL1B*, *TNF*, *SERPINE1*, and *IL18* genes. The number on the left of each sequence represents the positions with respect to the start of the transcription or TSS. Nucleotides in the box are the sequences selected for each gene. CpGs underlined and highlighted in bold are those that were quantified by MassARRAY EpiTYPER. Transcription start site (TSS). Coding DNA sequence (CDS). Supplementary Figure 2: relative cell viability (measured by MTT assay) after THP-1 monocyte incubation with folic acid (11.3 *μ*M), choline (105 *μ*M), vitamin B_12_ (18.5 nM), and methyl donor mixture. Data are shown as the means ± SD (*n* = 6). Differences between groups were analyzed by one-way ANOVA.
