# Supplementary material for: Folic Acid Improves the Inflammatory Response in LPS-Activated THP-1 Macrophages
Source: Mediators Inflamm. 2018 Jul 4;2018:1312626. doi: 10.1155/2018/1312626 (PMC6079441; doi:10.1155/2018/1312626)
Supplement: Supplementary Materials — Supplementary Table 1: primer sequences used for MassARRAY EpiTYPER assay. Supplementary Figure 1: genomic localization and nucleotide sequences of CpG sites covered by the MassARRAY EpiTYPER probes for the study of DNA methylation levels of IL1B, TNF, SERPINE1, and IL18 genes. The number on the left of each sequence represents the positions with respect to the start of the transcription or TSS. Nucleotides in the box are the sequences selected for each gene. CpGs underlined and highlighted in bold are those that were quantified by MassARRAY EpiTYPER. Transcription start site (TSS). Coding DNA sequence (CDS). Supplementary Figure 2: relative cell viability (measured by MTT assay) after THP-1 monocyte incubation with folic acid (11.3 μM), choline (105 μM), vitamin B12 (18.5 nM), and methyl donor mixture. Data are shown as the means ± SD (n = 6). Differences between groups were analyzed by one-way ANOVA. [file 1312626.f1.pdf]

Supplementary table 1. Primer sequences used for MassArray Epityper assay.

Gene	Primer	Sequence	Length (pb)	CpG covered
IL-1β	left	5'-gtgtttgtagttttagttgttggg-3'	331	6
	right	3'-tctcttaataataccaaccaaaaattatca-5'		
TNF-α	left	5'-tttggtttttaaaagaaatggaggt-3'	273	8
	right	3'-tccttaataaaaaaacccataaactca-5'		
SERPINE1	left	5'-tttggtataaaaggaggtagtggtt-3'	343	12
	right	3'-actctcctacaatcacccctaaaac-5'		
IL18	left	5'-tttgttgagtttttgttttttgg-3'	251	6
	right	5'-cctctaattaccataactaactttcca-3'		

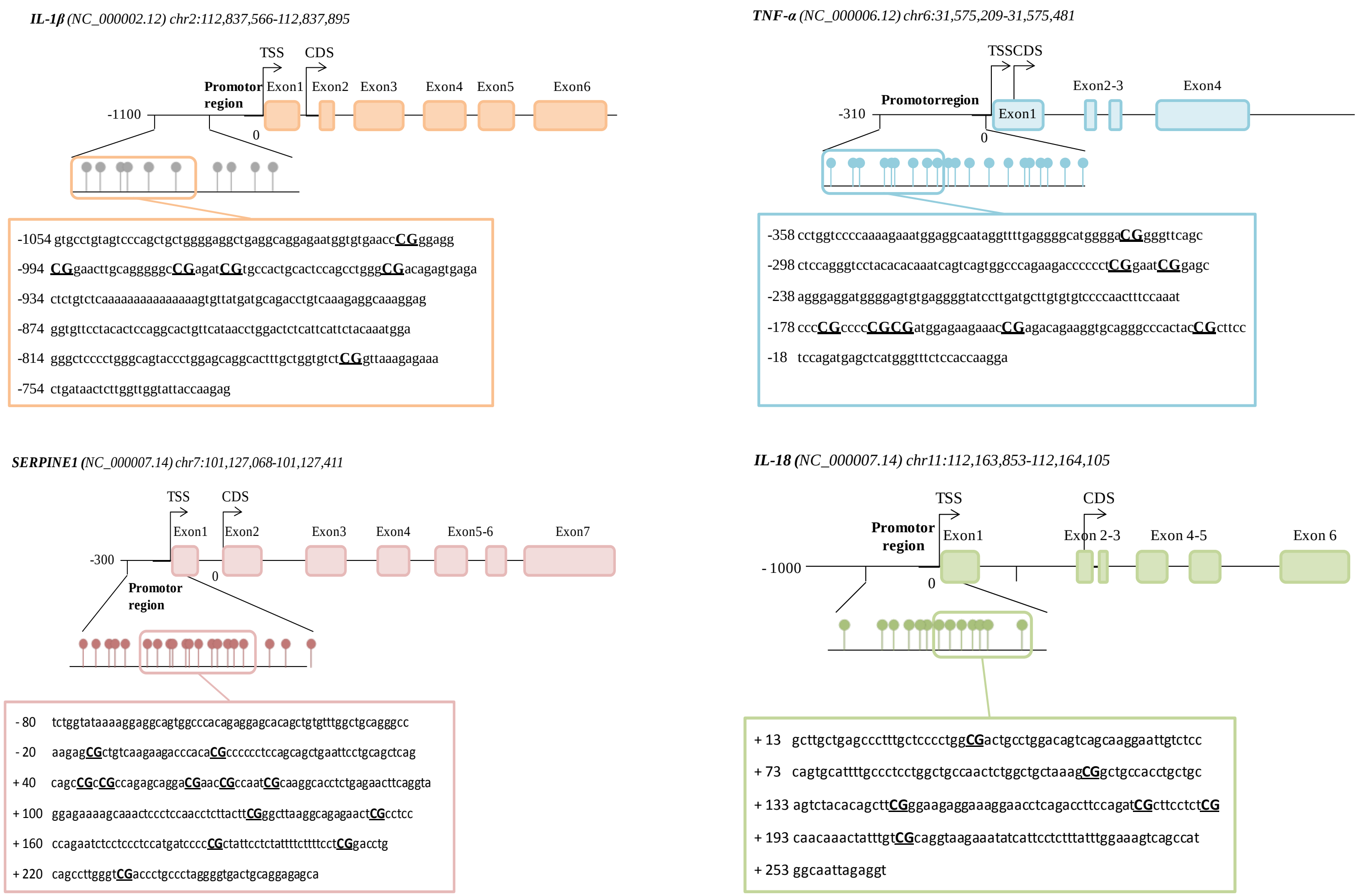

Supplementary figure 1. Genomic localization and nucleotide sequences of CpGs sites covered by the MassARRAY EpiTYPER probes for the study of DNA methylation levels of *IL1B*, *TNF*, *SERPINE1* and *IL18* genes. Number of the left of each sequence represents the positions with respect to the start of transcription or TSS. Nucleotides in the box are the sequences selected for each gene. CpGs underlined and highlighted in bold are those that were quantified by MassARRAY EpiTYPER. Transcription Start Site (TSS). Coding DNA Sequence (CDS).

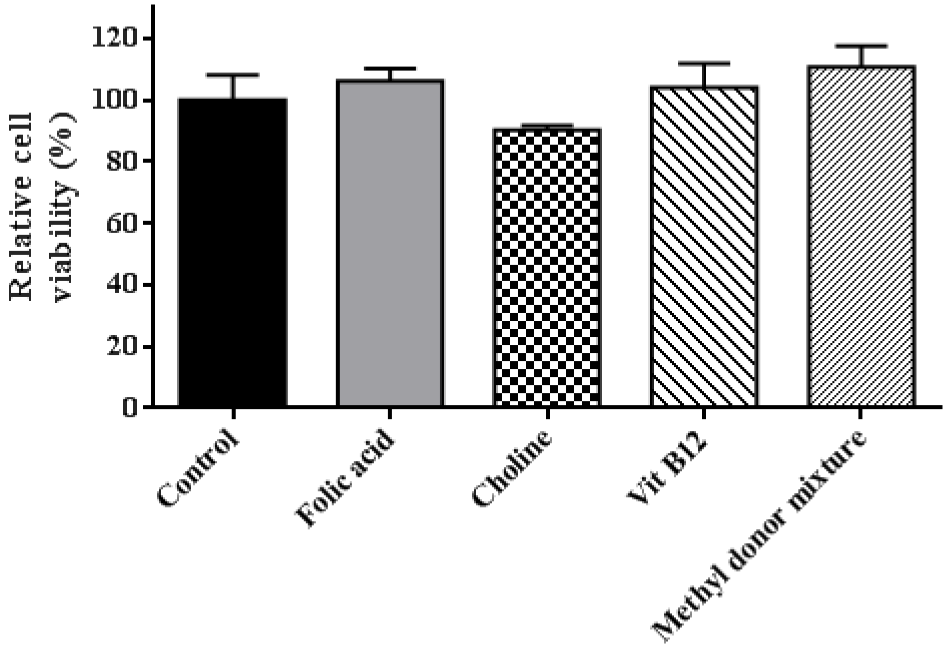

Supplementary Figure 2. Relative cell viability (measured by MTT assay) after THP-1 monocyte incubation with folic acid (11.3 μM), choline (105 μM), vitamin B12 (18.5 nM) and methyl donor mixture. Data are shown as the means ± SD (n=6). Differences between groups were analyzed by one-way ANOVA.
